# 

**Published:** 1887-08

**Authors:** 


### The Chicago Medical Journal and Examiner.

**Vol. 55. No. 2.**

### CONTENTS OF THIS NUMBER.

**Original Communications—** Page

Penetrating Gunshot Wound of the Abdomen. E. Andrews ... 125
Gunshot Wound of the Abdomen, with Perforation of the Intestines. F. T. Andrews ... 127
Gonorrhœal Peritonitis. J. E. Greene ... 128
Transverse Fracture of the Patella J. Y. Brown ... 131

**Foreign Correspondence—**

London Letter ... 135
Chiavari Letter ... 137

**Editorial—**

Ninth International Medical Congress ... 139
Tyrotoxicon in Food ... 139
Bacteria and Tetanus ... 140

**Reports of Medical Societies.**

The Mississippi Valley Medical Association ... 141

**Abstracts and Extracts—**

Obstinate Case of Constipation ... 142
Dyspepsia of Infants ... 142
Paralytic Phenomena in Simple Psychosis ... 143
Causes of Subcutaneous Inflammation and Suppuration ... 145
The Abuse of Mineral Waters ... 147
The Pupil in its Semeiological Aspects ... 148
Forms of Albumen in the Urine, and their Tests ... 151
Angina Pectoris without Lesion of the Coronary Arteries ... 156
Dyspeptic Origin of Visual Troubles ... 156
Retention of Urine ... 157
The Treatment of Intra-Peritoneal Injuries ... 160
Interference with Locomotion as a Uterine Symptom ... 162
The Rheumatic Element in Chorea of Early Childhood ... 165
Mercurialism in Lying-in Women Undergoing Sublimate Irrigation ... 166
The Therapeutic Uses of Cocaine ... 167
Grafting of Human Bone ... 169
The Action of Hydrochlorate of Hyoscine ... 170
Morbid Anatomy of the Fallopian Tubes ... 171
Pathology of the Laryngeal Vagus ... 173
Intestinal Obstruction from Impaction of a Gall-Stone in the Ileum ... 174
Climate as a Therapeutic Agent in Phthisis ... 178
A New Remedy for Burns ... 183

**Book Reviews and Notices** ... 184

